# Safety and effects of a home-based Tai Chi exercise rehabilitation program in patients with chronic heart failure: study protocol for a randomized controlled trial

**DOI:** 10.3389/fcvm.2023.1237539

**Published:** 2023-11-29

**Authors:** Qian Jiao, Chao Meng, Haoqiang He, Shanshan Li, Fan Xu, Weilu Cui, Yuqing Lou, Zining Li, Jing Ma, Di Sun, Huidong Wu, Haixia Li

**Affiliations:** ^1^ Department of Cardiology, Guang'anmen Hospital of Chinese Academy of Chinese Medical Sciences, Beijing, China; ^2^ Graduate School, Beijing University of Chinese Medicine, Beijing, China; ^3^ Department of Medical Examination, Guangdong Second Provincial General Hospital, Guangzhou, China

**Keywords:** Tai Chi exercise, aging, chronic heart failure, cardiac rehabilitation, randomized controlled trial

## Abstract

**Introduction:**

Chronic heart failure (CHF), as the final stage of the progression of many cardiovascular disorders, is one of the main causes of hospitalization and death in the elderly and has a substantial impact on patients' quality of life (QOL). Exercise-based cardiac rehabilitation (CR) has been shown to considerably enhance QOL and prognosis. Given the barriers to center-based CR faced by most developing countries in the form of expensive instruments, the development of home-based CR is necessary. Tai Chi, as an instrument-free exercise, has been shown to be successful in treating elderly CHF individuals. Fu Yang, as one of the academic concept of Traditional Chinese Medicine (TCM), believes that the fundamental pathogenesis of CHF is the gradual decline of Yang, and emphasizes the restoration of Yang physiological function in the treatment process. Therefore, we develope a home-based Tai Chi exercise rehabilitation program called Fu Yang Tai Chi (FYTC) for elderly CHF patients by combining the Fu Yang Theory of TCM with the CR theory. The objective of this study is to evaluate the effectiveness, acceptability, and safety of the program.

**Methods and analysis:**

We suggest conducting a parallel randomized controlled clinical trial with open label. Eighty CHF elderly participants will be randomly assigned in a 1:1 ratio to the FYTC rehabilitation program group or the moderate-intensity aerobic walking control group. Eligible participants will engage in either three sessions weekly of FYTC or walking exercise for 12 weeks. The primary outcome is the relative change in 6 min walk distance (6MWD). The secondary outcomes are the plasma levels of N-terminal pro-B-type natriuretic peptide (NT-proBNP), QOL, left ventricular ejection fraction (LVEF), left ventricular end-diastolic diameter (LVEDd), self-rating anxiety scale (SAS) and depression scale (SDS), exercise skills, and noninvasive hemodynamic monitoring. Throughout the trial, adverse events will be recorded for safety evaluation. Researchers who are blinded to the treatment allocation will analyze the data.

**Ethics and dissemination:**

This research was authorized by the Guang'anmen Hospital Ethics Committee of the Chinese Academy of Medical Sciences (2022-141-KY). Our findings will be shared online and in academic conferences as well as in peer-reviewed journals.

**Trial registration number:**

ChiCTR2200063511.

## 1. Introduction

Heart failure (HF) is the leading cause of death in elderly individuals. According to epidemiological surveys, the mortality rate was 7.2% and readmission rate was 31.9% for patients with chronic heart failure (CHF) (1). The incidence of HF increases with age (2). The prevalence of heart failure among Chinese people aged 80 years or older is 12%, and advanced age has become an important factor for poor prognosis in patients with heart failure (3). Different physical and mental comorbidities and symptoms, such as fatigue, depression, anxiety, edema, shortness of breath due to the chronic and prolonged disease course, and therapeutic processes have a serious and negative impact on the quality of life (QOL) of CHF patients, resulting in lower QOLs compared with healthy individuals and other patients with chronic illnesses (4). Lower QOL correlates with increased hospitalization times and mortality rates and higher costs imposed on health systems, families, and patients (5). Although guideline-directed therapies are known to improve patient outcomes in CHF (6), few pharmacologic therapies have been demonstrated to improve quality of life, which is associated with subsequent hospitalization and mortality (7).

Cardiac rehabilitation (CR) is an essential component of chronic heart failure management (8). Increasing studies have shown that reasonable exercise rehabilitation training can improve elderly heart failure patients' exercise tolerance, cardiopulmonary function and quality of life as well as prognosis (9). Such exercise rehabilitation training is an important way to maintain the independent living ability of the elderly (10, 11). Despite this, the majority of eligible heart failure patients in both developed and developing countries fail to participate in cardiac rehabilitation (12). Reported impediments to access include lack of transportation, considerable out-of-pocket expenses, scheduling conflicts, and a dearth of specialized rehabilitation centers and facilities (13). The number of patients participating in cardiac rehabilitation has declined even more markedly as measures intended to block the spread of coronavirus disease 2019 (COVID-19) have exacerbated these barriers (14). Notably, the best way to overcome these barriers may be to facilitate home-based exercise rehabilitation. In recent studies it has been found that home-based CR exercises are neither less safe nor effective than center-based CR exercises (15). The results of current studies have demonstrated that patients who underwent home-based exercise rehabilitation had positive improvements in their exercise ability, quality of life, and compliance. Additionally, there is a decrease in events such as coronary revascularization, cardiac mortality and readmission. However, studies on home-based cardiac rehabilitation programs for elderly individuals are very limited (16). Therefore, there is an urgent need to find an exercise rehabilitation program suitable for older chronic heart failure patients, one that will improve their cardiac function and exercise tolerance, thereby improving their quality of life.

Tai Chi is a mind-body exercise with medium-intensity aerobic exercise and safe for the elderly. Tai Chi is commonly considered as a type of exercise that involves an active mindful element, which means it focuses on training profound inwardly mental attention in addition to the habitual bodily movements involved in traditional forms of exercise (17). Many clinical studies have proven that Tai Chi exercise is beneficial for improving cardiopulmonary exercise function (18), preventing falls (19), preserving vascular endothelial function (20), reducing dyslipidemia, and regulating blood sugar and blood pressure (21). Tai Chi is thought to be a viable complement to exercise-based cardiac rehabilitation in chronic heart failure patients (22, 23). Fu Yang, as one of the academic concept of Traditional Chinese Medicine (TCM), believes that the fundamental pathogenesis of CHF in the elderly is the gradual decline of Yang, and emphasizes the restoration of Yang physiological function in the treatment process. According to Yin/Yang Theory of TCM, functional activities of the body are classified as Yang. In order to simplify and improve the original Tai Chi and make it easier for the elderly to master, we combine the Fu Yang Theory of TCM with the theory of cardiac rehabilitation and develope a home-based Tai Chi exercise rehabilitation program called Fu Yang Tai Chi (FYTC) for elderly patients with CHF.

However, there are no clinical trials to confirm the effectiveness and safety of the exercises; therefore, we designed a single-center, open label, randomized controlled trial (RCT) to study the effects of “Fu Yang Tai Chi” as an adjunctive therapy on the quality of life of elderly patients with CHF. If the results are positive, this therapy could become a good way for older people with CHF to exercise at home that is also affordable.

## 2. Methods and analyses

### 2.1. Study design and recruitment

**Figure 1:**
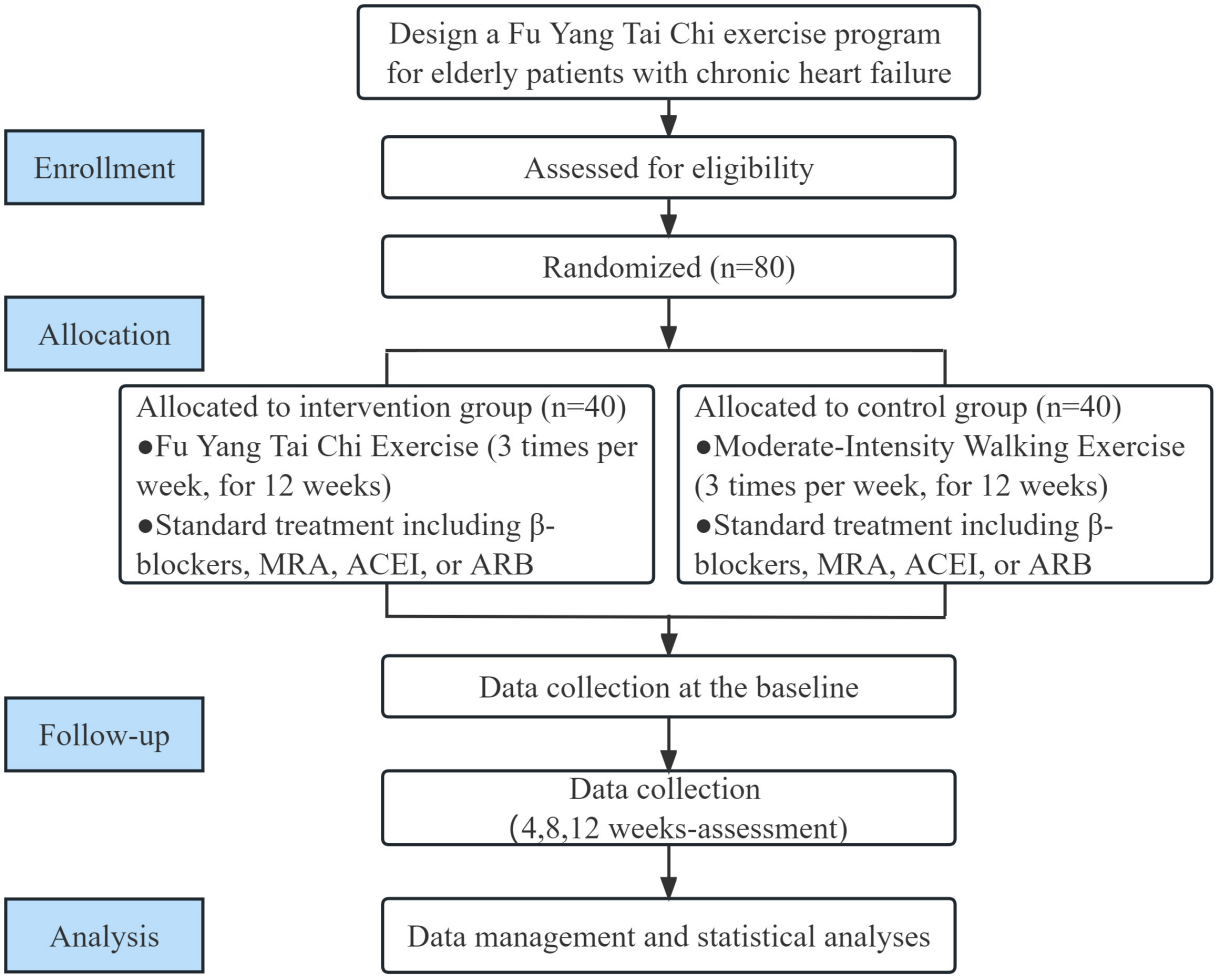
The flowchart used in the template is from CONSORT 2010.

The Guang'anmen Hospital of the Chinese Academy of Traditional Chinese Medicine serves as the recruitment site for the participants and the single center for this open, randomized controlled trial. A total of 80 volunteers will be enlisted after providing written informed consent. All of the prospective patients will be given comprehensive information regarding the trial in detail, covering topics such as its objectives, procedures, and intervention approaches as well as its potential benefits and drawbacks. Throughout the 12-week testing period, we will randomly assign participants in a 1:1 ratio to either the Fu Yang Tai Chi exercise group or the walking control group. The outcomes will be assessed at 12 weeks (immediately after the intervention). The study design process is shown in the study flow chart in Figure 1. The protocol for this clinical trial complies with the Standard Protocol Items: Recommendations for Interventional Trials (SPIRIT) (24) and follows the the Consolidated Standards of Reporting Trials (CONSORT) (25). The study is currently recruiting patients and is expected to be completed by 31 December 2023. All assessments including at baseline (Week 0), at each visit (Week 4 and Week 8) and immediately after completion of the rehabilitation program (Week 12) will be undertaken at the hospital.

### 2.2. Participants

#### 2.2.1. Inclusion criteria

(1) Aged between 60 and 75 years old;
(2) Fulfilling the diagnostic criteria for heart failure with reduced ejection fraction (HFrEF) included in the 2021 ESC Guidelines for the Diagnosis and Treatment of Acute and Chronic Heart Failure;
(3) New York Heart Association functional classification (NYHA class) II and III;
(4) Currently taking part in less than 60 min of physical exercise per week;
(5) Learned about the clinical trial programs and voluntary participants and provided written informed consent.

#### 2.2.2. Exclusion criteria

(1) Limited range of motion in the extremities;
(2) Inadequately managed hypertension (systolic blood pressure ≥180 mmHg or diastolic blood pressure ≥100 mmHg);
(3) Resting heart rate over 120 beats per minute, or complicated by malignant arrhythmia (Atrial fibrillation, Ventricular fibrillation, Ventricular tachycardia and more);
(4) Patients who suffer from a serious infection, chronic obstructive pulmonary disease, respiratory failure, or pulmonary cardiac disease;
(5) Patients with severe primary illnesses, including tumors and hematopoietic system disorders;
(6) Severe mental illness or cognitive dysfunction;
(7) Combined severe metabolic and hormonal disorders (such as thyrotoxicosis and diabetic ketosis);
(8) Recent treatments by modern or traditional Chinese medicine rehabilitation methods in the past 3 months.

### 2.3. Randomization and concealed allocation

Following the completion of the baseline evaluation, qualified participants will be assigned to either the FYTC exercise group or the walking control group in a ratio of 1:1 using a table of random numbers that is created by the statistical program SPSS 25.0. The grouping information will be maintained by an independent statistician who is unaware of the participant recruiting, evaluation, and intervention processes. The statistician will give the list to the research coordinator after placing it in opaque, sealed, sequentially numbered envelopes. The research coordinator will open one envelope at a time after screening to receive a random number and information about participant grouping.

### 2.4. Blinding

Both patients and personnel were unaware of the group the patient would be allocated to at the time of performing the baseline 6 min walk test. It is impossible to maintain blinding of trial participants and exercise instructors due to the nature of the intervention. However, researchers assessing primary outcome assessment will be masked for group assignment.

### 2.5. Intervention

Participants in both groups will receive home-based cardiac rehabilitation. Eligible participants will be randomly assigned to the FYTC exercise group or the walking control group, and an individualized exercise prescription will be formulated. Patients in both groups will be asked to exercise at least 3 times per week for 12 weeks. Exercise intensity will be monitored using Borg's Rating of Perceived Exertion (RPE) scale or adjusted using the heart rate reserve method based on the HR max determined during the initial 6 min walk test. The 6 min walk test will be conducted at the hospital. During the process of home-based CR we will equip each subject with a heart rate detector, and the monitoring will be conducted remotely through WeChat video and the heart rate detector. Exercise sessions in both groups will consist of warm-up, exercise, and relaxation periods. In addition, an exercise diary will be provided to all participants to record their daily physical activities over the trial period.

#### 2.5.1. FYTC exercise group

**Figure 2:**
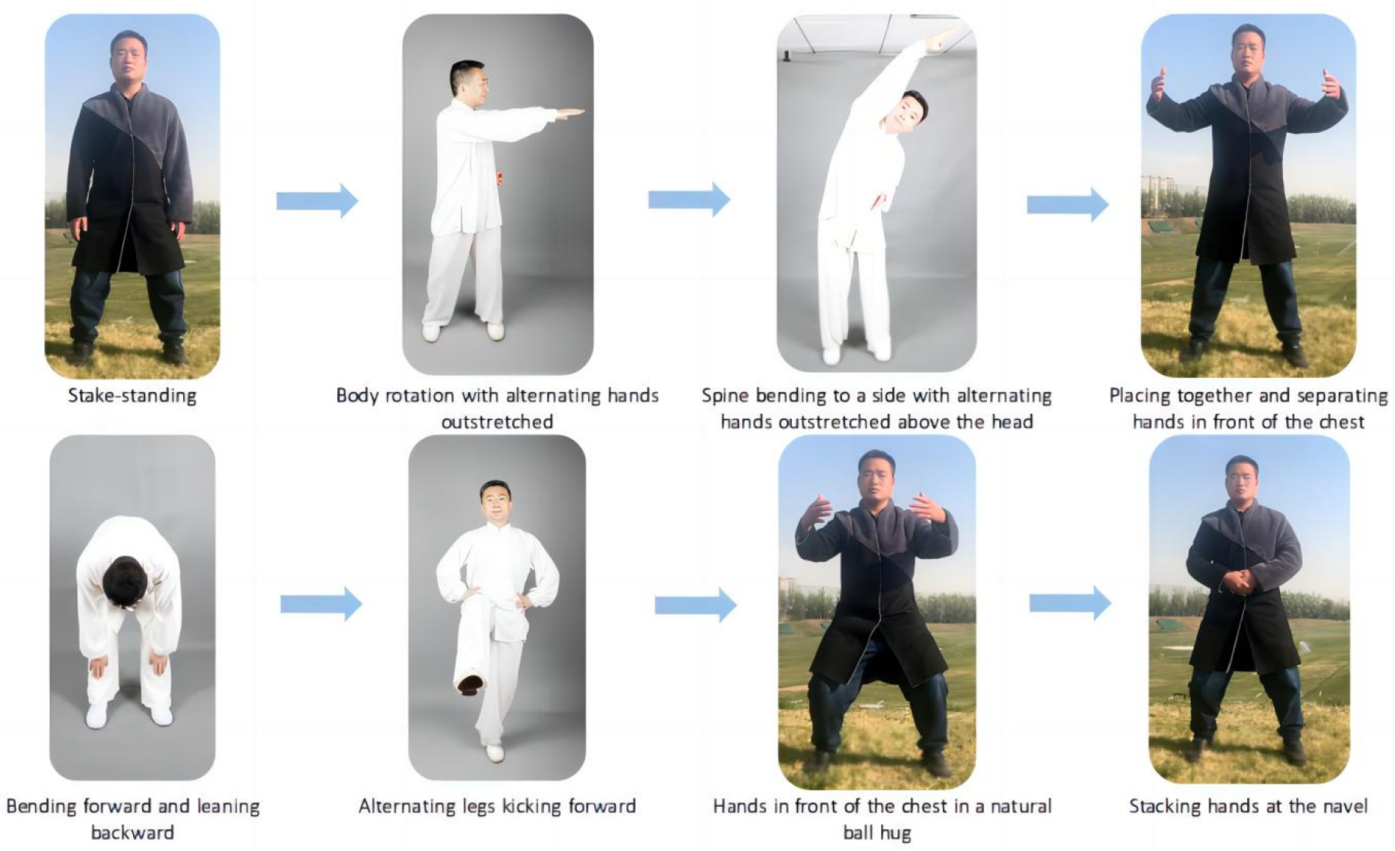
Exemplary Fu Yang Tai Chi; the portrait in the photo is correctly taken; the fuglemen are the home-based Tai Chi cardiac rehabilitation program creators. Figure 2 illustrates the eight methods that constituted Fu Yang Tai Chi.

The FYTC exercise group will practice FYTC at home with remote guidance training by two professional Tai Chi instructors. Each exercise routine will last at least 50 min (including a 10 min warm-up, 30 min FYTC core movements, and 10 min of relaxation) three times a week for a 12-week period. The FYTC exercise core workout (30 min) will consist of the following eight movements (Figure 2): stake-standing, body rotation with alternating hands outstretched, spine bending to a side with alternating hands outstretched above the head, placing together and separating hands in front of the chest, bending forward and leaning backward, alternating legs kicking forward, hands in front of the chest in a natural ball hug, and stacking hands at the navel. We will formulate a standardized program of movements and record the video for participants to learn. Regulating the breath, regulating the body (physical activity) and regulating the mind (mental regulation) will be included in Tai Chi warm-up and relaxation exercises. These movements are excellent for relieving stress, calming the mind, and facilitating complete physical and mental relaxation.

In the continuous training, the Borg RPE range of 11–13 will be used to regulate exercise intensity and energy expenditure, while the patient's heart rate will be monitored by a heart rate monitor. All participants will be instructed to maintain their usual activities and refrain from new strength training. Video review of the WeChat software and individual feedback from subjects will be checked frequently for accuracy and compliance in an effort to ensure that participants perform each movement. We will ask all patients to go about their daily activities and refrain from attempting any novel forms of strength training.

#### 2.5.2. Walking control group

Participants in the control group will walk at a moderate intensity three times weekly at consistent for a total of twelve weeks. Each routine will consist of three components: a 10 min warm-up, a 30 min session of moderate-intensity walking exercise, and a 10 min rest period. Participants will be guided and supervised by a professional cardiac rehabilitation coach throughout the intervention. The Borg RPE range of 11–13 or 40%–80% of reserve heart rate will be used to regulate exercise intensity and energy expenditure. Our cardiac rehabilitation program will be customized for each participant and closely supervised. Each participant who take part in the study will receive careful monitoring in a cardiac rehabilitation program that will be specifically designed for them. To achieve moderate intensity exercise, exercise will be progressively increased in duration and intensity.

### 2.6. Concomitant treatment

Each participant will receive the standard of care treatment as determined by their doctor in accordance with clinical recommendations (26, 27), such as diuretics, beta-blockers, mineralocorticoid receptor antagonists (MRAs), angiotensin-converting enzyme inhibitors (ACEIs), or angiotensin receptor blockers (ARBs). On the Case Report Form (CRF), the exact date and reason for any changes to medications will be written down.

### 2.7. Outcomes

#### 2.7.1. Primary and secondary outcomes

The items to be measured and the window of time for data collection are listed in Table 1. Changes in cardiorespiratory endurance as evaluated by the 6 min walk distance (6MWD) were the primary outcome of interest.

**Table 1:** Study process of the trial.

	Baseline period	Intervention period		
Study phase time	Visit 1	Visit 2	Visit 3	Visit 4
	−7 to 0 days	4 weeks	8 weeks	12 weeks
Informed consent	√			
Inclusion/exclusion criteria	√			
Allocation	√			
Demographic data	√			
Previous history, medical history, and allergies	√			
Comorbidities and comedications	√			
Safety assessment	√	√	√	√
6 min walk distance	√	√	√	√
MLHFQ score	√	√	√	√
SAS and SDS scores	√	√	√	√
NT-proBNP	√	√		√
Echocardiographic measurements	√	√	√	√
Exercise skills	√	√	√	√
Noninvasive cardiac output monitoring	√	√		√

The secondary outcomes will be as follows:

(1) Variations in N-terminal pro-B-type natriuretic peptide (NT-proBNP) plasma levels;
(2) Improvement in quality of life (QOL). Improvement in QOL will be evaluated using the Minnesota Living with Heart Failure Questionnaire (MLHFQ) and the Pittsburgh Sleep Quality Index (PSQI). The MLHFQ which is the most commonly used scale for measuring quality of life in CHF patients, will be utilized to evaluate how these two exercise regimens affected patient quality of life from a physical, emotional, social, and psychological standpoint. A self-assessment questionnaire called the PSQI (28) rates sleep disruptions and quality. It has five questions for the bed partner's or roommate's peer answer and 19 questions for the subject's self-reaction (if one is available). The index, which ranged from 0 to 21, will be calculated by adding the scores from seven different categories. A score of 0 denotes the absence of any sleep disturbances or excellent sleep quality, whereas a higher number denotes unsatisfactory sleep.
(3) Alterations in self-rating anxiety scale (SAS) and self-rating depression scale (SDS) scores;
(4) Improvement in left ventricular end-diastolic internal diameter (LVEDd) and left ventricular ejection fraction (LVEF) echocardiographic measures;
(5) Enhancement of exercise abilities: Exercise abilities include dexterity, balance, and grip strength. An instrument made by CAMRY (product model EH101) called a grip strength dynamometer will be used to test grip strength; it measures the maximal isometric muscular strength of the hand and forearm muscles. We will independently measure the time of one-foot standing with eyes closed and one-foot standing with eyes open for the balancing test (29), confirming the Romberg experiment. Three tests will be administered to measure the participants' times until they lost their balance. The best measurement result out of the three will be chosen. The seated forward flexion test will be used to measure flexibility.
(6) Changes in noninvasive cardiac output monitoring (NICOM): NICOM is a noninvasive hemodynamic monitoring technique based on bioelectrical impedance (30, 31). The intravascular blood flow varies when the heart expands and contracts, and corresponding variations in the impedance of current across the chest also occur (32). The principle of thoracic impedance is precisely implemented in NICOM to enable assessment of cardiac function and myocardial contractility by processing the parameters obtained from the calculus hemogram of cardiac impedance. Myocardial contractility parameters include the velocity index (VI), acceleration index (ACI), pre-ejection period (PEP), systolic time ratio (STR), and left ventricular ejection time (LVET). Cardiac function parameters include cardiac output (CO), cardiac index (CI), stroke volume (SV), stroke volume index (SVI), left cardiac work (LCW), and left cardiac work index (LCWI).

#### 2.7.2. Safety outcomes

All study participants will receive safety monitoring during the intervention, focusing on vital signs (temperature, heart rate, respiration, and blood pressure), laboratory testing (standard blood tests, liver and kidney function tests), and adverse events (AEs), which will be recorded throughout the trial. Exercise injury, falls, and drug withdrawal symptoms (such as tiredness, headache, discomfort, lack of strength, dizziness, and dyspnea) will be included in the AEs (33). The relation of each adverse event to the intervention will be determined and documented on the adverse event CRF over the trial period. Additionally, the Human Research Committee will be quickly informed of any negative incidents. The 6 min walk test will be only performed on individuals who are qualified and capable of finishing it. The appropriate intensity and duration of exercise will be modified in accordance with risk stratification for enrolled patients who fit the requirements for rehabilitation training. Researchers will inform participants with chronic heart failure about rehabilitative exercise, including guidelines about when it would not be a good idea to train and what exercises should be done.

### 2.8. Sample size

The size of the sample is determined by comparing the projected improvement in 6MWD between the walking control group and the FYTC exercise group. In the current study (34), the means and their SDs (mean ± SD) of the 6MWD in the control and intervention group were (289 ± 165, 412 ± 116). At least 60 patients will be required for the experimental and control groups each given considerations of a type-I error rate of 0.05, a power of 90% (type-II error rate of *β* = 0.1). Considering a possible withdrawal rate of 20%, the total number of patients required will be 76. To facilitate statistical and clinical collection, the sample size will be expanded to 80.

$$n_{1}={\left[\frac{(\mu_{\alpha }+\mu_{\beta })\sigma }{\delta }\right]}^{2}\frac{\left(1+\kappa \right)}{\kappa },n_{2}=\kappa n_{1}$$

In this equation, *κ* stands for the ratio between the two sample cases (*κ* = 1), *δ* for the expected improvement in the six-minute walk trial, and *σ* for the SD.

### 2.9. Statistical analyses

Third-party statisticians who will be blinded to the group assignment and intervention method carried out the statistical analysis. For missing data, we use regression modeling or average interpolation for missing data prediction, avoiding as much as possible the impact of missing values on the analysis results. The intention-to-treat (ITT) concept will be used to analyze primary or secondary outcomes. Data analysis will be performed using the statistical program SPSS 25.0 (SPSS Inc., Chicago, USA). For non-normal distributions, the median and IQR will be used to represent continuous variables, while counts or percentage will be used to describe categorical variables. Fisher's exact probability approach or the chi-square test will be used to define categorical variables. Continuous variables that conform to a normal distribution will be described by mean and SD. Student's *t* test with two sample will be used to characterize continuous variables with normal distributions. A modified *t* test will be employed when the variance between the groups with a cutoff of 0.05. In cases where the data do not have a normal distribution,the Mann-Whitney *U* test will be used to examine intragroup or intergroup variations before and after therapy.

If necessary, We will perform multivariate analyses using multiple linear regression to account for adjustments for possible confounders covariates selected on the basis of clinical relevance and univariate outcomes. All *P* values under 0.05 will be regarded as indicative of statistical significance for all tests, which are all run in both directions.

### 2.10. Data management and monitoring

Two cardiologists will constitute the independent Data and Safety Monitoring Committee (DSMC), which is responsible for monitoring trial data (including spotting any patterns, such as an increase in accidents) and, if needed, suspending the experiment. Before the trial began, all of the researchers, including research assistants, investigators, doctors, and statisticians, participated in unified preclinical trial training to ensure that they had a comprehensive understanding of the trial protocol, standard study protocols, and their respective responsibilities. For instance, research assistants will encourage volunteers to exercise at home and complete activity diaries. Additionally, all researchers will receive training in outcome evaluation, data management, and consistent case report form (CRF) completion. Regular gatherings will be set up to discuss trial-related issues and choose the best remedies. The DSMC statistician will also compare the data between the *p*-CRF and the electrical CRF, and any changes to the data will be carefully tracked and documented.

### 2.11. Trial status

The trial is still ongoing. Enrollment began in October 2022 and is expected to end in December 2023.

## 3. Discussion

This study explore the impact of home-based Tai Chi exercise on clinical outcomes in individuals who suffered from chronic heart failure. Safety concerns will be also taken into consideration. Having a high morbidity and mortality rate, CHF is a severe issue everywhere, particularly in societies with aging populations, and it places a heavy physical and psychological burden on patients (35, 36). The effectiveness of CR, particularly exercise-based CR, in elderly CHF patients has been supported by a vast body of research (37, 38). Regardless of the training method, structured exercise training has been found to enhance prognosis and quality of life in CHF patients (39). COVID-19 blocking strategies have led to challenges to the center-based rehabilitation model, and a new investigation of home-based rehabilitation has emerged to establish a new paradigm for cardiac rehabilitation (40). With home-based and center-based CR delivering equivalent advantages in terms of clinical and health-related quality of life (41, 42) for the same cost, it is vital to research the discovery of optimum CR activities that are both the most useful and easiest to do.

As a set of Tai Chi exercises designed specifically for elderly patients with chronic heart failure, Fu Yang Tai Chi exercise retains the essence of traditional Tai Chi and starts from a holistic model of tuning the body, mind and breath. It also discards the drawbacks of aerobic exercise and emphasizes the importance of spiritual care in moderate exercise, realizing the combination of “exercise prescription” and “psychological prescription.” The whole set of movements is even and slow, combining movement and stillness, and is a unique connotation of aerobic physical and mental exercise.

The trial will use a rigorous random allocation method and blind to evaluators and statistical analysts to reduce bias. Therefore, the study is expected to produce reliable results. Four limitations of the current research should be acknowledged. In comparison to previous multicenter comprehensive investigations, the current research's sample size is modest, and it is a single-center study. It is unknown whether other locations and ethnic groups may show comparable results. Second, it is challenging to keep track of the research participants' increased physical activity. Although all participants will be requested to keep a record of their regular physical activity or exercise, this is insufficient to adequately gauge their daily activity levels. Third, this experiment is only followed up for 12 weeks. However, follow-up time should be suitably increased to clarify long-term clinical results and the possible effects of home-based Tai Chi training.
